# Comparative Ophthalmology

**Published:** 1887-01

**Authors:** L. Webster Fox

**Affiliations:** Philadelphia, Pa.


### Art. V.—COMPARATIVE OPHTHALMOLOGY.

### CLINICAL OBSERVATIONS.

BY L. WEBSTER FOX, M. D.
*Philadelphia, Pa.*

Catarrhal Ophthalmia.—(Pink-Eye.)—During an epidemic of influenza rife among the horses, in and about Philadelphia several years ago, many hostlers, drivers, and cabmen came under my observation, suffering from catarrhal ophthalmia, with a history that this disease was contracted while handling horses suffering with "pink-eye," as it was vulgarly termed. Extending my researches into this field of comparative ophthalmology, I elicited many interesting clinical facts, and, at the same time alleviated much suffering, not only in human beings but in animals, by the proper application of certain remedies.

As to the infection of the disease which always acccompanied the influenza, there was no doubt. It was observed that where one horse became afflicted, his neighbor would in a short time show evidence of the infection. There being many avenues through which the cocci could be conveyed, especially when horses are crowded together, as of necessity they are, in large stables, that it is impossible to enjoy immunity against contagion. Conjunctivitis is much more common among horses than would be supposed by the casual observer. Müller, in the *Berliner Poliklinik*, found, among 3784 diseased animals 160 suffering from conjunctivitis, a very high percentage when it must be remembered that animals suffering from conjunctivitis are rarely entered at such an institution for such a disease. In man, the disease was local, only affecting the conjunctiva, while in the animal, the mucous membranes of the eye and nostrils were alike affected, with accompanying symptoms of chill and fever.

The disease in horses, dogs, and cows is preceded by a period of photophobia, lachrymation, swelling of the lids, yellowish discoloration of conjunctiva and iritis. In from ten to twenty-four hours these symptoms grow worse, involving not only the cornea, but the uveal tract as well. Schindelka found in several cases keratitis parenchymatosa, and uveitis. Vogel found the conjunctiva swollen to such a size that the lids could not be closed. These conditions are only found in the aggravated forms of the disease. From 24 to 36 hours after the first symptoms manifest themselves, we have profuse muco-purulent discharges from eyelids and nostrils. Upon close inspection we find the conjunctiva of the lids swollen, congestion and engorgement of the capillaries, at times subconjunctival hemorrhages, the cornea steamy, pupil contracted, and reacting to light feebly, the ocular folds much swollen, while the orbital tissues became infiltrated.—(Dieckerhoff.) These symptoms are usually accompanied by pain, loss of appetite and drowsiness. By watching the eye symptoms, we can judge of the progress of the disease, as they continue throughout the fever; an improvement in the ocular symptoms is indicative of convalescence.

It may be interesting from a bacteriological point to mention that the disease does not assume that same degree of virulency in man. Whether the secretions from the conjunctiva of man do not form as productive a medium for the full development of the micro organisms, remains for future investigation. But the secretions produced in man by the *cocci* from the horse reproduced themselves when applied to a healthy conjunctiva, as I have had several families under treatment where but one individual was affected primarily, he in a short time communicating the disease, by the aid of a basin, to other members. In the treatment of the disease it falls to my province to deal with ocular symptoms only. The swelling of the lids must be relieved, as well as the discharges stopped as soon as possible. The continued pressure producing keratitis, which may end in *ramollissment* of the cornea, followed by prolapse of iris or iritis, and by choroiditis, ending in loss of vision. The eye should be bathed frequently with warm poppy fomentations, and the following collyrium applied to the eye-ball four times daily with a small syringe:

| | | | |
|---|---|---|---|
| ℞. | Zinci chlor. | | grs. iv. |
| | Hydrarg bichlor. | | grs. $\frac{1}{30}$ |
| | Aq. camph. | | |
| | Aq. destil. | ā ā | ℨ ii. |
| ℳ. | | | |

In keratitis complicated with iritis, atropia sulph. grs. iv. to ℥ i. must be applied to eye-ball three times daily.

Choroiditis.—A valuable horse was recently brought under observation with the following history: The keeper noticed a haziness of the right cornea, congestion of the sclerotic blood vessels, and the vision apparently disappearing. In two months the cornea of the left eye assuming the same condition. At this time I made an examination of both eyes. The right eye was totally blind. The cornea clear, but iris bound to the capsule of the lens, which was cataractous. The left cornea was steamy, and iris discolored; after dilating the pupil, the ophthalmoscope revealed marked choroiditis, which was recognized by the marked disturbance of the tapetum. The vision was very much lessened, as the animal was clumsy and would walk against objects. Owing to the iritis and choroidal change an unfavorable prognosis was given.

The treatment instituted was atropia sulph. grs. iv to ℥i, to dilate and rest the, iris while 40 grs. of potass. iodidi, three times daily was given internally. An issue was also placed in the skin, at the angle of the jaw. The treatment was continued for two weeks with marked benefit, at this time the vision had improved, and the acute symptoms had disappeared. The owner since informing me that the animal could avoid obstacles with comparative ease. From the uninterrupted train of symptoms, this case suggests itself as being one of sympathetic ophthalmitis; the energetic treatment preventing total loss of vision. The ophthalmoscope which aided in the discovery of the above disease should never be omitted in certain obscure visual defects in animals, although the field of this department cannot be as extended and useful as in man, yet all students of veterinary medicine should give it their careful consideration, bearing in mind, however, that the fundus oculi of different animals presents a different picture, on account of a different distribution of the arterial and venous system, as well as the deposit of pigment. That of man and the pig are alike, yet that of the horse and cow differ from that of man.

In using the ophthalmoscope we have two methods, the *direct* and *indirect*. In our experience we have found the indirect the most useful. Its practical application is as follows: we use a 9-inch concave mirror, of about 2½ inches diameter and placing a light in front or above the animal's head we reflect into the pupil an image of the flame, while the examiner looks through the perforation in the center of the mirror, standing about 16 inches away, we interpose a + sph. 2-inch lens before the eye, resting the little finger on the brow; the aerial inverted image is now formed of the fundus oculi. The brilliant reflex from the tapetum varying with the color of the animal.

Recently,* Drs. Lang and Barrett, of London, published an interesting article on *The Refractive Character of the Eyes of Mammalia.* While the authors lament that their results can only be regarded as approximate in character, yet they must be congratulated upon furnishing statistics which are highly interesting to all students of comparative ophthalmology. Their mode of examination was by retinoscopy, the light being thrown into the fundus oculi by means of a mirror with a focal distance of 25 c.m. If the fundus reflex traveled with the mirror, the animal was myopic (near-sighted), if the reflex moved in an opposite direction the animal was hyperopic (far-sighted).

The examinations by the direct method were unreliable and impracticable. Of 185 eyes examined, 24 were emetropic

*The Royal London Ophthalmic Hospital Reports, July, 1886.

(normal for distant vision), 145 hyperopic, or hyperopic and astigmatic (far sighted), 13 myopic, or myopic and astigmatic (near sighted), 3 mixed astigmatism. Owing to the large per cent. (7.) of myopic eyes the authors pre-suppose another factor in its development than that engendered by sendentary habits or the use of the accomodating muscle. To prove this in a measure, the accommodation of a cat was thoroughly paralyzed by atropia, he was, however, able to make a speedy capture of a mouse with accuracy, proving the existence of very little accommodation in this animal. Among the animals examined were rabbits, guinea-pigs, mice, rats, cows, horses, cats, and dogs. From the above figures the authors think that as an optical instrument, the eye of the horse, cow, cat and rabbit, is superior to that of the rat, mouse, and guinea-pig.

